# Research on the interface design of ASD children intervention APP based on Kano-entropy weight method

**DOI:** 10.3389/fpsyt.2025.1508006

**Published:** 2025-02-25

**Authors:** Bingchen Zhang, Yunlin Qi, Yuling Yang, Jiping Zhang

**Affiliations:** ^1^ Department of Industrial Design, School of Mechanical and Electrical Engineering, Jiangsu Normal University, Xuzhou, China; ^2^ Department of Automotive and Transportation, Yancheng Polytechnic College, Yancheng, China

**Keywords:** Kano-entropy weight method, ASD children, demand analysis, intervention APP, interface design

## Abstract

**Background:**

In recent years, there has been a notable increase in the prevalence of Autism Spectrum Disorder (ASD) among children in China. To enhance the efficacy of ASD intervention apps and streamline the design process for designers, this study proposes an interface design research method for ASD intervention apps based on the Kano-entropy weight method.

**Methods:**

First, the basic research process for ASD children is extracted by combining the characteristics of the Kano model and the entropy method. Additionally, representative app samples currently available on the market are collected and organized. Subsequently, the representative needs of ASD children are subjected to analysis and synthesis. Secondly, the data obtained from the questionnaires are organized, and the entropy method is employed to calculate the weight of the need indicators. Subsequently, the characteristics of the intervention apps and the magnitude of the weight values are employed in the analysis of different categories of needs. The case design practice is conducted with a focus on strengthening the effectiveness of the intervention apps from a human-computer interaction perspective, with a particular emphasis on relevant ASD children’s intervention training. The study employs the Kano-entropy weight method to analyze the demand indicators for ASD intervention apps in terms of content, usability, and visual design through survey analysis.

**Results:**

This study compiled 26 pieces of user demand information, extracted 17 specific indicators, and classified them into three types: content-based, operable, and visual. The interface design of the intervention app focuses on improving emotional abilities. Further enrich the content of the ASD children’s intervention app, improve its operation, enhance visual appeal, provide reference and basis for subsequent related designs, and improve the effectiveness of intervention training.

**Conclusions:**

The objective is to enhance the efficacy of the apps from the perspective of human-computer interaction. This endeavor seeks to furnish pertinent theoretical references for the design of navigation interfaces for intervention apps and to provide effective assistance to educators and designers.

## 1 Introduction

Autism Spectrum Disorders (ASD) is a neuro developmental disorder that occurs in the early stage and is characterized by social interaction, communication disorders, narrow range of interests, and stereotyped behaviors (1). Missing early intervention will endanger the healthy growth of children. With the formulation and improvement of special education regulations, the intervention training of ASD children has gradually changed from teacher-led to independent learning (2). Intervention APP as a visual means under the new technology, has been widely used in the field of autism rehabilitation, providing a high-efficiency, low-cost effective way for the rehabilitation of ASD children (3). The study found that children with ASD have obvious advantages in visual cognition, and the interface design of intervention APP based on user demand analysis can better adapt to the cognitive characteristics of children with ASD, providing new ideas for early intervention training and education issues (4, 5). Appropriate APP intervention interfaces can enhance children’s adaptability to different educational apps and have a positive impact on their cognitive and innovative abilities (6, 7). Children exhibit significant visual cognitive differences in various drawing styles composed of different lines, symbols, and colors during infancy (8). Zhong Guangming et al. (9) used the KANO model to summarize the demand indicators for different levels of satisfaction in the design of playable children’s seating, and further studied and concluded that3A product requirement item. Zhu Yunfeng (10) used Kano evaluation results to screen and classify the functional attributes of multi-functional children’s beds for families with two children, clarifying the priority of users’ functional requirements. Deypir (11) proposed a simple and effective APP security risk measurement method based on entropy weight method. The risk score is positively correlated with the malware infection rate, providing effective judgment for users to install APPs. Zhu (12) et al. combined the entropy weight method with the relevant contextual information features of mobile apps to construct an app classification model, and verified that this classification method can effectively and efficiently improve the performance of mobile app classification. However, how to organically integrate the three and use the Kano entropy weight method to analyze user needs still needs further improvement, which is also the main content of this study.

## 2 Related research

### 2.1 Research status of ASD children’s intervention APP interface

APP is the abbreviation of Application Program, which refers to third-party applications installed on mobile terminal devices that provide users with various convenient services (13). As a result of the development of information demand in the information society, APP has developed rapidly in the field of rehabilitation teaching for autism in recent years, and has been widely used in related rehabilitation training (14). Silva G F (15) trained children with ASD on collaboration and social skills through an app, and the results showed that they can significantly improve their social interaction skills. Fage C et al. (16) used mental interpretation software to intervene in the emotional regulation ability of ASD children in their research, and found that compared with traditional behavior training programs, their emotional self-regulation ability and social adaptation ability have been significantly improved. Law G C (17) found that the use of APP to assist children in language intervention training can effectively improve the use of spontaneous vocabulary and gestures in children with ASD.

The APP interface is the main carrier for users to obtain information and an important entrance to realize their own needs. The quality of its design can directly affect the user’s visual experience and operating experience (18). The design of the ASD children’s intervention APP interface aims to meet the needs of ASD children, improve their satisfaction with the intervention APP interface, and then improve the intervention effect. Bozgeyikli et al. (19) studied the impact of changes in different APP interface properties on the operating experience of ASD children, and found that they are more interested in interface design with low visual fidelity and animation instructions, and have a better operating experience. Boste et al. (20) used alternating therapy design to explore the differences in the intervention effects of different APP interfaces on children with ASD, and the results showed that attractive and guiding interfaces can effectively improve their communication skills.

**Figure 1:**
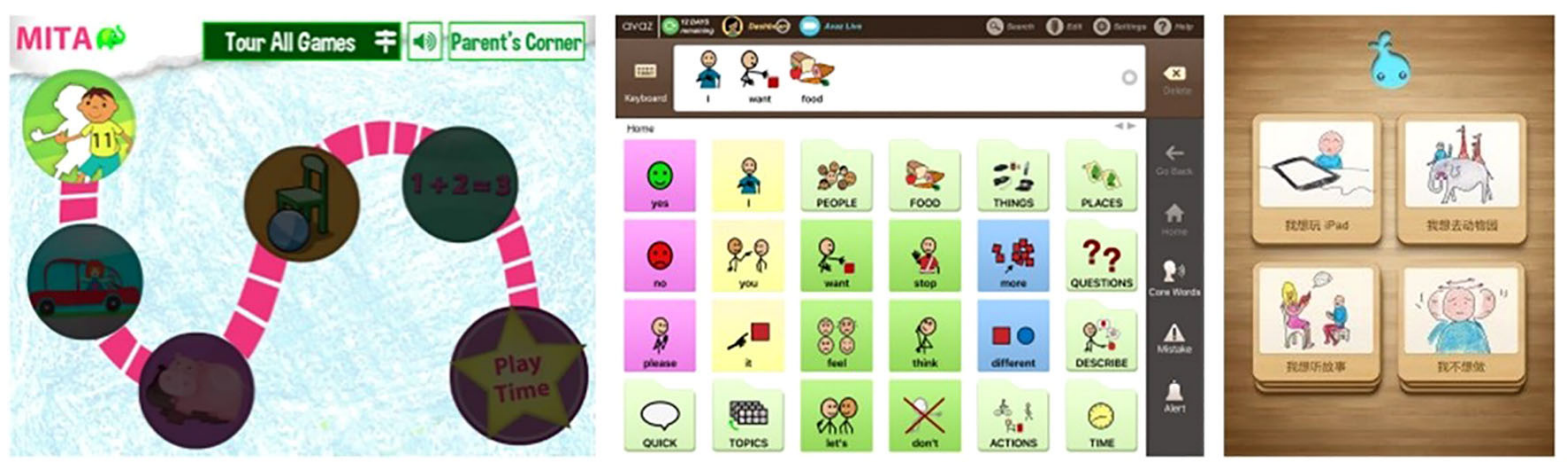
Interface symbols in hand drawn style of the “Little Raindrop” APP.

“Autism Therapy with MITA” developed by ImagiRation LLC is the first and only speech therapy application supported by clinical data. The APP designed the interface into a fun roadmap mode to arouse children’s curiosity and desire to explore. The failed levels are in the state to be lit, and ASD children are encouraged to move forward. The buttons use circular symbols to achieve a simple and clear visual effect, deeply loved by children with autism and parents (see **Figure 1** on the left). “Avaz” is an enhanced and alternative communication application that allows ASD children to have their own voice. It has been launched in more than 20 countries, and has won awards in the United States, Finland, Sweden and India. The combination of patterns and text in the APP interface is clearly organized and distinguished by simple background colors, which is highly indicative. The use process makes ASD children feel happy (see **Figure 1**). “Little Raindrop” developed by Professor Wei Liping from the Institute of Life Sciences of Peking University is currently the most downloaded and most popular ASD children intervention app in China. The palace format interface is designed to intuitively display various content. The abstract hand-drawn style graphic symbols are vivid and interesting, which are in line with children’s cognitive psychology, and are convenient for ASD children to choose and express their inner needs (see **Figure 1** on the right). It can be seen that excellent APP interface design plays an important role in increasing the value of APP. Arpita (21) found through experiments that in various learning activities such as copying and classroom response, the developed APP provides more effective intervention training for children with autism, and children are more actively seeking the advantages and disadvantages of the learning process during training. Therefore, it is particularly important to introduce the visual cognitive characteristics of ASD children into APP interface design.

### 2.2 The principle and application of Kano model

**Figure 2:**
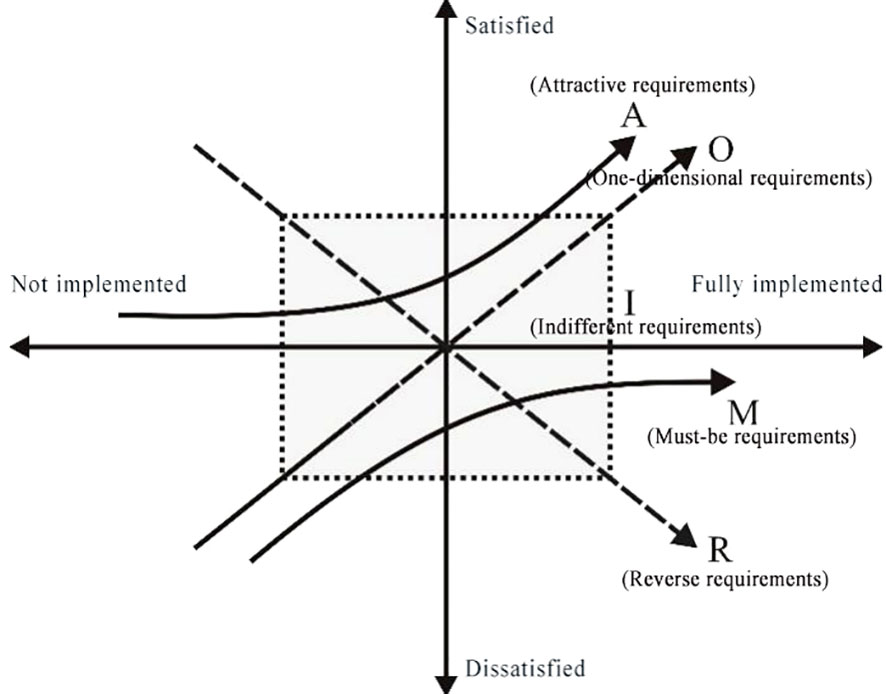
Kano model.

Inspired by Herzberg’s two-factor theory, Tokyo Institute of Technology professor Noriaki Kano published a paper “Charm Quality and Essential Quality” in 1984, and constructed a Kano model in this paper. This model can help designers understand user needs at different levels, classify and prioritize user needs, and then find out important factors that satisfy users and improve product design quality (22). The Kano model defines user requirements for products into five levels, including: Must-be requirements, One-dimensional requirements, Attractive requirements, Indifferent requirements and Reverse requirements. This model breaks through the simple linear relationship between user demand types and satisfaction, as shown in **Table 1**; **Figure 2** (M represents basic demand, O represents expected demand, A represents excited demand, I represent irrelevant demand, and R represents reverse demand).

**Table 1:** Requirement type definition table.

Demand type	Definition
Must-be requirements	When this demand is optimized, user satisfaction will not increase; when this demand is not provided, satisfaction will be greatly reduced.
One-dimensional requirements	When this demand is provided, user satisfaction will increase; when this demand is not provided, user satisfaction will decrease.
Attractive requirements	When this demand is provided, satisfaction will be greatly improved; when this demand is not provided, user satisfaction will not decrease.
Indifferent requirements	When this demand is provided, user satisfaction will not change; when this demand is not provided, satisfaction will not change.
Reverse requirements	The user does not have this requirement, and the user satisfaction will be reduced after the provision.

As a tool for analyzing and planning quality and user satisfaction, Kano model has been widely used in product design, interaction design, service experience design and other fields (23–25). Yuan Hao et al. (26) applied the Kano model to the functional requirements analysis of the interactive interface of washing machines, and provided an effective reference for the optimal design of the interactive interface. Ginting (27) used the Kano model to calculate the importance of website interface requirements, accurately grasped user needs, and improved and innovatively designed the website interface. Gong Lei (28) used the Kano model to analyze the impact of nursing process improvement on the satisfaction of children and parents, and determine key improvement elements.

Yang Xibo (29) used the fuzzy Kano model to explore the functional requirements of children’s dining chair design, providing a reference for improving user stickiness. Wang Juan (30) constructed a demand priority model for preschool children’s sensory experience play teaching aids based on the Kano model. Yu Guangchen (31) applied the Kano model to the functional requirements analysis of children’s intelligent monitoring products. Li Jing (32) analyzed user key needs through the Kano model and constructed a service system design scheme for outdoor fitness products. Zhao et al. (33) studied the functional design indicators of modular storage products for children using a comprehensive Kano model and Analytic Hierarchy Process.

### 2.3 Entropy method

Entropy weight method refers to the objective weighting method that determines the weight of indicators according to the degree of variation of the observed values of various indicators. It can find the hidden regularity between indicators from a large amount of information and effectively avoid the deviation caused by subjective factors. Tian Bo et al. (34) used the entropy method and AHP to analyze the influencing factors of APP user privacy information leakage, construct a reasonable evaluation index system, and provide effective reference for various platforms, departments and users. Liu X et al. (35) used the entropy method to determine the weight coefficients of various indicators in the reliability evaluation of the human-computer interaction interface, which improved the direction and ideas for the design and innovation of the interaction interface. Wang Yixuan (36) used the entropy method to establish the evaluation model of the music mobile APP interface, and quantitatively analyzed various evaluation indicators, which provided an effective reference for the optimization design of the APP interface. Hassan (37) combined the entropy method with the Kano model to establish a product life cycle assessment system to clarify vague user needs.

Fu Han (38) used entropy weight method to effectively obtain the multi-level needs of the elderly population in the design of aging friendly interfaces for medical apps. Huang Jinsong (39) used entropy weight method to rank the importance of children’s functional requirements for hand washing products. Zhao Chen (40) used the entropy weight method to determine the functional requirement hierarchy of cinema seat design based on user contextual experience, effectively improving user satisfaction. Deng Zhao (41) applied the entropy weight method to the functional design of shared emergency robots, improving the accuracy and scientificity of the design process. Li Saisai (42) used the entropy weight method to prioritize the design requirements of the ward logistics robot service system.

## 3 Research process

**Figure 3:**
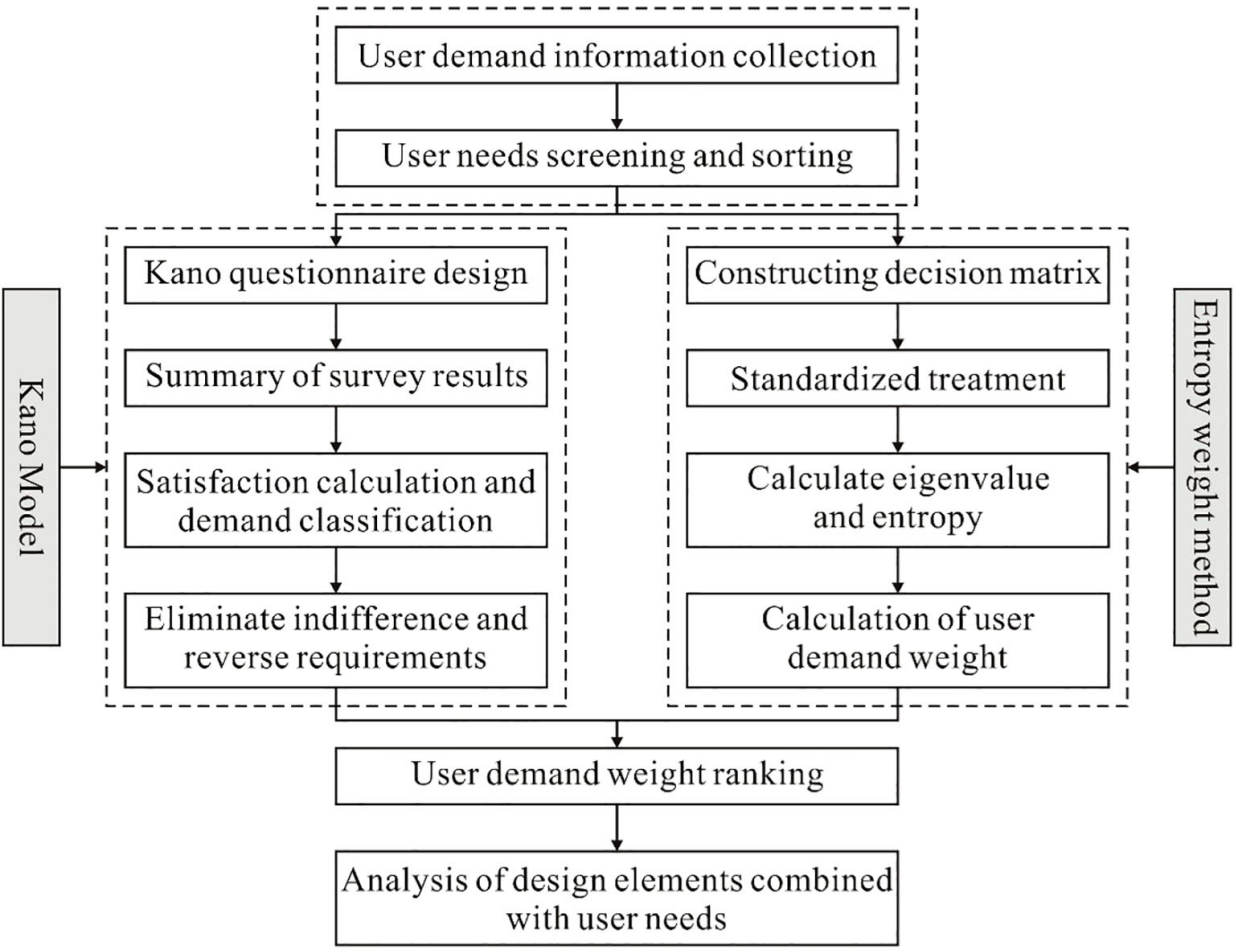
Research process.

According to literature analysis, it is found that combining the Kano model with the entropy method and applying it to the design and research of the ASD child intervention APP interface can weaken the subjectivity of the Kano model when processing demand indicators, and use the entropy weight method to quantify the important indicators of demand. To improve the accuracy and credibility of the research, the research process is shown in **Figure 3**.

### 3.1 Obtain user demand information

**Figure 4:**
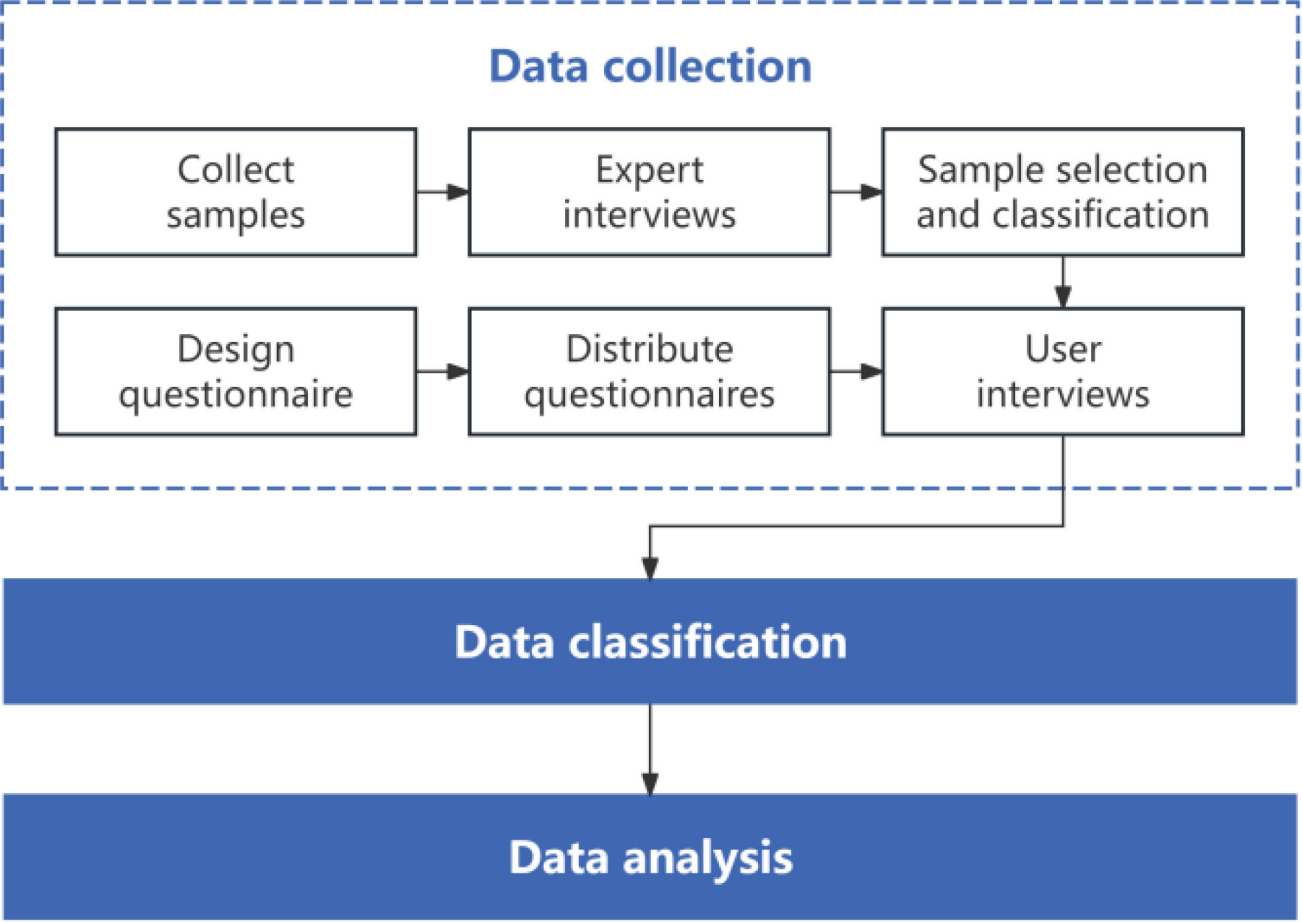
Data collection program diagram.

User needs are multi-level, including physical, psychological and subjective feelings, and there are big differences. User needs analysis is the investigation and study of user needs in the early stages of product design and development. Its purpose is to create higher product value and enhance user experience. There are four common ways to acquire user needs: user interviews, questionnaires, usability testing and data analysis. The main process of collecting user demand related data in this study is shown in **Figure 4**.

### 3.2 Classification of user needs based on Kano model

The classification of user needs based on the Kano model mainly includes 4 steps: Kano questionnaire design, user needs sorting, satisfaction coefficient calculation and needs classification, and elimination of indifferent needs and reverse needs.

1) A two-factor questionnaire design is adopted, that is, each demand/function question consists of two sub-questions, positive and negative, which are the responses of users when they have or do not have a certain demand. The user has five options when answering the question, namely: like very much, like, no opinion, dislike, dislike very much.

2) According to the survey results of the Kano questionnaire, combined with the needs classification table, the user needs are sorted and summarized, as shown in **Table 2** (M represents Must-be demand, O represents One-dimensional demand, A represents Attractive demand, I represent Indifferent demand, and R represents Reverse demand). Must-be requirements is the basic requirement of users for products. When product features do not meet user needs, user satisfaction will decrease; When its features meet user needs, users may not feel satisfied because the product performs well. One-dimensional requirements are a requirement that is proportional to the degree of satisfaction of the user’s needs. If such needs are met or performed well, user satisfaction will significantly increase. Attractive requirements are a requirement that is not excessively expected by users, but once it is met, the satisfaction level of users is also very high. On the contrary, even when expectations are not met, users will not show obvious dissatisfaction as a result. Indifferent requirements refer to the fact that regardless of whether such features exist in the product, they have no impact on the user’s usage process. Reverse requirements refer to the phenomenon where user satisfaction decreases after a product provides its functionality, and the degree of provision is inversely proportional to the level of user satisfaction.

**Table 2:** Demand classification table.

		Have a need				
		like very much	like	no opinion	dislike	dislike very much
**Does not have a need**	like very much	——	A	A	A	O
	like	R	I	I	I	M
	no opinion	R	I	I	I	M
	dislike	R	I	I	I	M
	dislike very much	R	R	R	R	——

3) When there are two equal maximum values when users classify their requirements, or the gap is small, using the “maximum value” method to determine the type of user requirements lacks rigor (43). In 1993, Berger (44) proposed that when the percentage difference of the user’s judgment of the demand index type is within 5%, this problem can be solved by calculating the ratio of the user satisfaction coefficient, as shown in Equations 1, 2 Shown.

Among them, $\;SI_{i}$ represents the relative satisfaction coefficient, that is, the impact on user satisfaction when the product has a certain demand; $\;DI_{i}$ represents the relative dissatisfaction coefficient, that is, the impact on user satisfaction when the product does not have a certain demand. $A_{i},M_{i},O_{i},I_{i}$ respectively represent the ratio of the user’s choice of this type of demand for the same demand question in the questionnaire survey.


(1) $$SI_{i}=(A_{i}+O_{i})/(A_{i}+O_{i}+M_{i}+I_{i});$$

(2) $$DI_{i}=-(M_{i}+O_{i})/(A_{i}+O_{i}+M_{i}+I_{i});$$

The ratio of user satisfaction coefficient  RI is the absolute value of $\;RI\;SI_{i}/DI_{i},$ and the value range of RI is shown in **Table 3** (45). This classification method can not only accurately classify user demand indicators, but also clearly understand the degree of impact on user satisfaction with or without a certain demand.

**Table 3:** Demand classification table.

$RI_{i}$ range	<^0^.^9^	^0^.^9^~^1^.^1^	>^1^.^1^
Demand type	M	O	A

4) Undifferentiated demand means that user satisfaction will not increase when the demand is provided, and user satisfaction will not decrease when the demand is not provided, that is, the user has an indifferent attitude toward this type of demand, and will not affect the overall design The impact should be eliminated; reverse demand means that the user does not have such a demand, but after providing it, it will cause a decrease in satisfaction, which will have a negative impact on the design, and should also be eliminated.

### 3.3 Use entropy method to calculate demand index weight

Using the entropy method to calculate the demand index weight mainly includes 4 steps: constructing a decision matrix, standardizing processing, calculating eigenvalues and entropy values, and calculating the demand index weight.

1) Invite m experts to use a five-level Likert scale (1~5: very unimportant, not very important, general, important, very important) to evaluate the importance of the n final selected demand indicators and construct a decision matrix ***B*** = $(b_{ij}{)}_{m\times n}$ ,the score of the i-th expert on the j-th demand index is represented by $b_{ij}$ .


$$B={\left(b_{ij}\right)}_{mxn}=\left[\begin{array}{cccc} b_{11} & b_{12} & \cdots & b_{1n}\\ b_{21} & b_{22} & \cdots & b_{2n}\\ \vdots & \vdots & \vdots \\ b_{m1} & b_{m2} & \cdots & b_{mn} \end{array}\right]$$

Since the higher the expert’s evaluation of the demand index, the more important the demand index is, so the demand index is judged to be a positive index, that is, the larger the index value, the better. Then use the positive correlation formula to standardize the decision matrix B, as shown in Equation 3.


(3) $${b^{\prime }}_{ij}=\frac{b_{ij}-\min b_{ij}}{\max b_{ij}-\min b_{ij}};$$

Use Equation 4 to calculate the proportion of the evaluation feature value of the i-th expert under the j-th demand index, that is, the proportion of the i-th expert’s evaluation of the j-th demand index, and get the matrix C= $(c_{ij}{)}_{m\times n}$ .


(4) $$c_{ij}=\frac{{b^{\prime }}_{ij}}{\sum_{j=1}^{n}{b^{\prime }}_{ij};}$$

Then calculate the entropy value E_j of the jth demand index, as shown in Equation 5.


(5) $$E_{j}=-k\sum_{j=1}^{n}c_{ij}\ln c_{ij},k=\frac{1}{\ln n};$$

4) From the entropy value of the jth demand index, the weight $W_{j}$ of the demand index can be calculated, as shown in Equation 6.


(6) $$W_{j}=\frac{1-E_{j}}{m-\sum_{j=1}^{n}E_{j}},0\leq W_{j}\leq 1,\sum_{j=1}^{n}W_{j}=1;$$

## 4 Case study

### 4.1 Collect experimental samples

**Figure 5:**
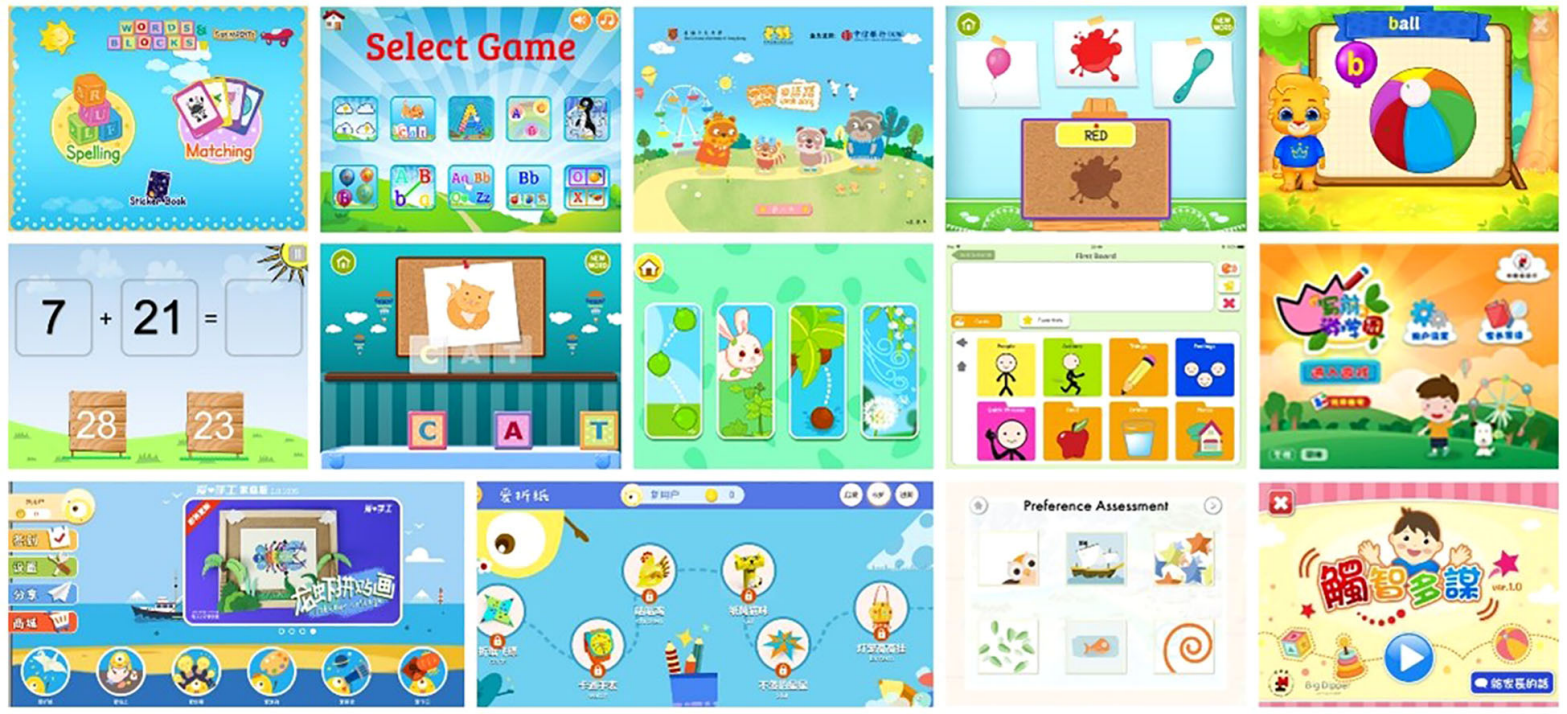
Part of the interface sample.

Through the APP Store, Huawei App Market, and Google App Market, samples of autism category apps are taken, combined with functional characteristics to classify the collected samples, and applications that cannot be used due to technical problems and whose content is obviously unrelated to ASD children are excluded. The samples of ASD children’s intervention APP were 28 cases of educational aids, 19 cases of language training, 15 cases of social skills, 13 cases of behavior intervention, 6 cases of puzzle games, a total of 81 cases (see **Figure 5**).

On the basis of preliminary screening, remove the interface pictures in apps with similar styles and low downloads, and invite 3 designers with senior children’s APP design experience and 2 graduate students majoring in visual communication design to combine the visual interface Based on comprehensive consideration of nature, guidance and market demand, 8 types of intervention APP interfaces were selected as typical samples. The main content information is shown in **Table 4**.

**Table 4:** Sample information introduction table.

Typical sample	APP name	The main function	Remarks
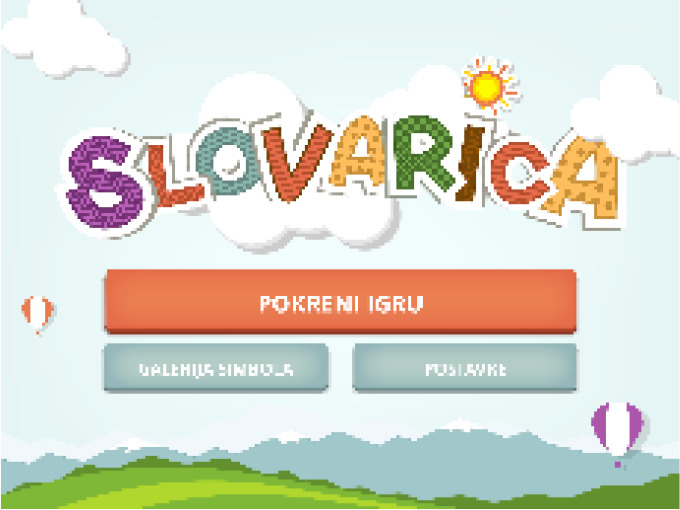	Slovarica	Help ASD children learn visual symbols and new phonetic forms, and improve their early literacy skills	Ranked 2nd in the IOS list and won many awards
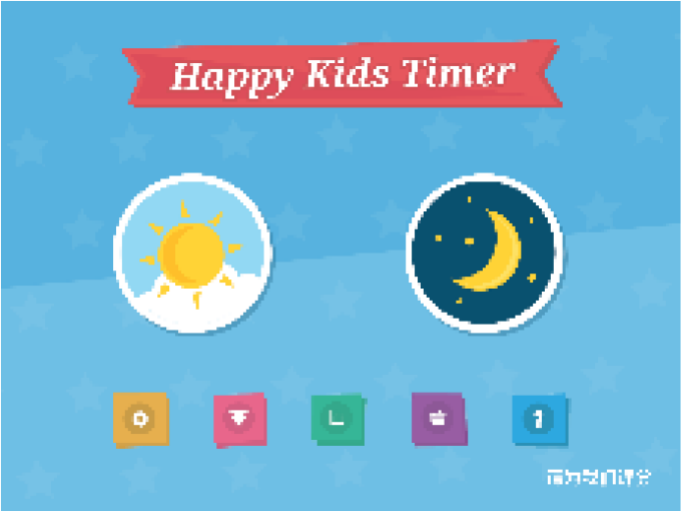	Happy Kids Timer	Use games to guide children to complete daily routines from morning to evening on time, acquire life skills, and cultivate independence	More than 300,000 people have used this app
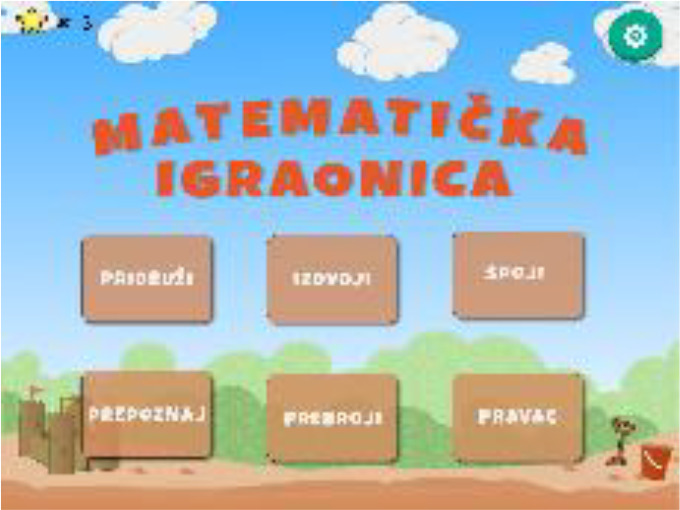	Mat igraonica	Help children master basic math knowledge and skills, including six number games: merge, separate, connect, check, count and run	Developed by the University of Zagreb, FER, focusing on the study of children with ASD for many years
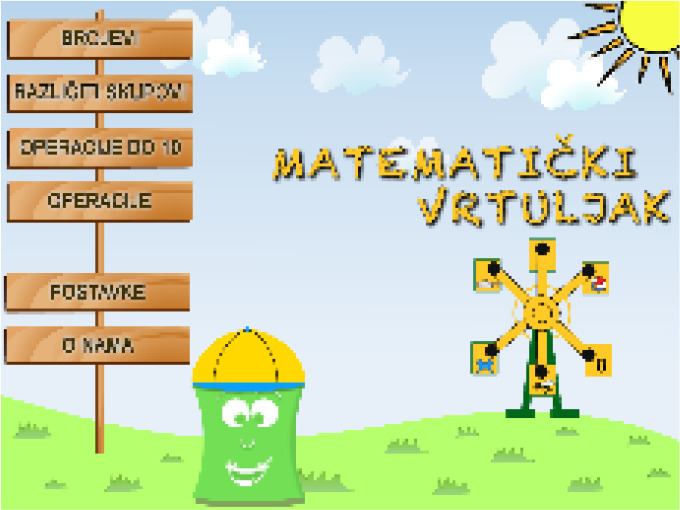	AAC Matemetika	Guide children to perform basic counting, set equality, and mathematical operations using numbers or symbols	Is currently the most downloaded math learning app on the market
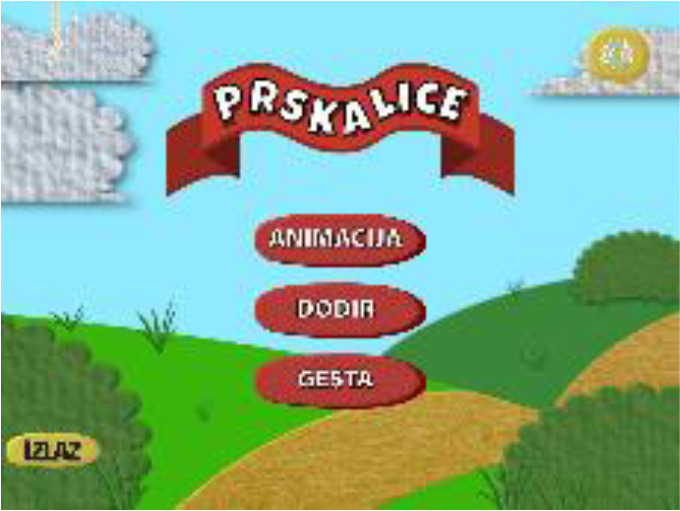	ICT AAC Prskalice	Help ASD children create and understand causality by presenting scenes in daily life	Developed by the University of Zagreb, FER, focusing on the study of children with ASD for many years
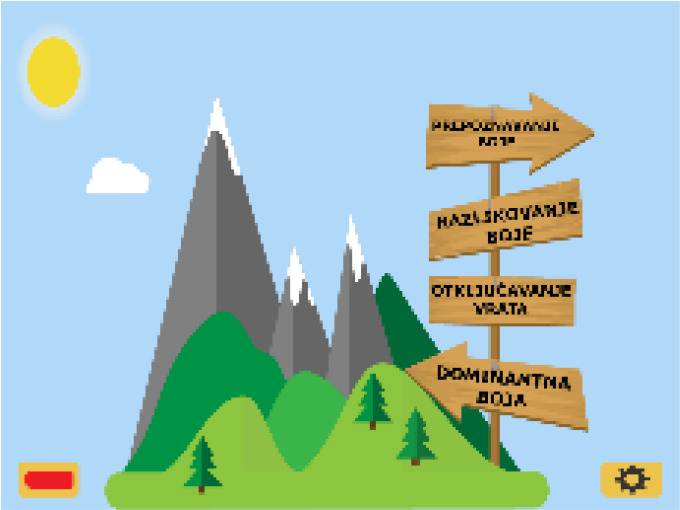	ICT AAC Ucimo boje	Use simple and clear audiovisual elements to provide a way to learn, identify and distinguish	Available in multiple languages, using global visual symbols to express information, with a wide range of applications
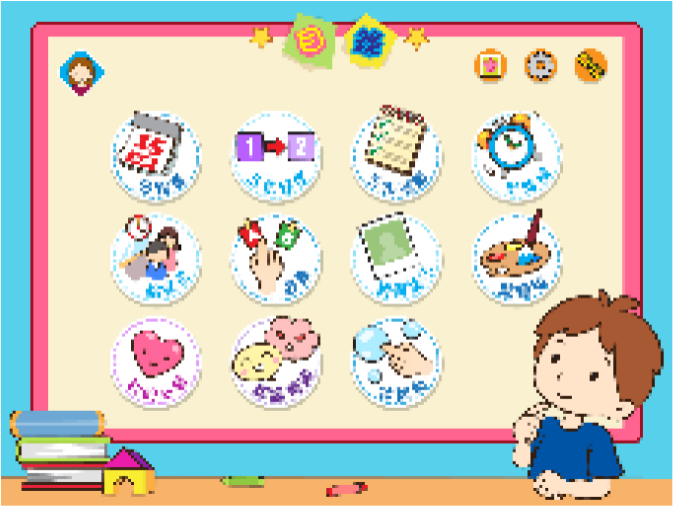	知情解意	Use visual cue tools to enhance the interest of ASD children in active communication and improve their ability to respond to difficulties	Developed by the team of Heep Hong Society, it is one of the largest children’s education and rehabilitation institutions in Hong Kong
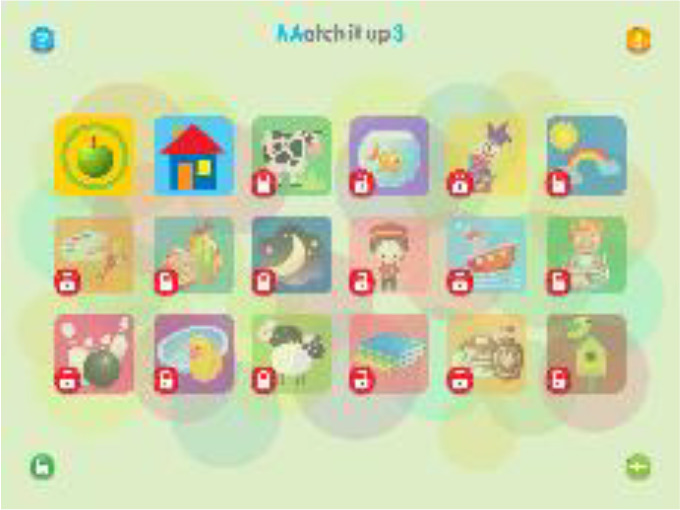	Match it up	Use children’s curiosity to improve cognitive and fine motor skills through matching games	Has been applied to more than 20 countries and is very popular among parents

### 4.2 Obtain information on children’s needs with ASD

Contact and invite 9 teaching assistants (all working ages over three years) from Xuzhou Herun Welfare Institute, Children’s Rehabilitation Hospital and Wucailu Children’s Development Center, and 3 designers with experienced children’s APP design experience to form expert group. Through the analysis of 8 types of typical intervention APP interface samples, combined with relevant literature review, and in-depth interviews with the expert group, the user needs of 26 intervention APPs were finally determined. The detailed information is shown in **Table 5**.

**Table 5:** Intervention in the collation of APP user demand information.

Numbering	Interface demand indicators	Numbering	Interface demand indicators	Numbering	Interface demand indicators
X_1_	Neat and reasonable	X_11_	Clear navigation	X_21_	Clear hierarchy
X_2_	Harmonious color matching	X_12_	Graphic symbols are lively	X_22_	Operation history
X_3_	Good operation feedback	X_13_	Rich character elements	X_23_	Beautiful icon elements
X_4_	Jump speed is moderate	X_14_	Family scene application	X_24_	Fun font form
X_5_	Concise and intuitive information	X_15_	Simple geometric shapes	X_25_	Subject style adjustment
X_6_	High content recognition	X_16_	Content attractive	X_26_	Personal platform push
X_7_	Voice interaction function	X_17_	The plot is coherent and reasonable	X_27_	Provide guide video
X_8_	Simple background elements	X_18_	Personalized function customization	X_28_	High degree of scene simulation
X_9_	Simple and convenient operation	X_19_	Various levels of content	X_29_	Two mode switching
X_10_	Vivid modeling	X_20_	Rich reward mechanism		

### 4.3 Classification of ASD children’s needs based on the Kano model

According to the user demand information in **Table 3**, the two-factor questionnaire design of the Kano model is carried out. Due to the weak language expression ability of ASD children, the subjects in this experiment are all experienced teaching assistants and parents (46). Select Xuzhou City Herun Welfare Institute, Wucailu Children’s Development Center and Children’s Rehabilitation Hospital to conduct questionnaire surveys. Before the survey, the content and meaning of each interface requirement index are introduced in detail to ensure the reliability and validity of the information obtained by the questionnaire. A total of 105 questionnaires were distributed and 87 were recovered, of which 82 were valid questionnaires, and the questionnaire effective rate was 78.1%. Combining **Tables 1**, **2** and Equations 1, 2 to classify user needs, the final questionnaire survey results are shown in **Table 6**.

**Table 6:** Questionnaire survey results.

Numbering	$A_{i}$ %	$M_{i}$ %	$O_{i}\%$	$I_{i}$ %	$R_{i}$ %	Satisfaction coefficient $\;SI_{i}$	Dissatisfaction coefficient $\;DI_{i}$	Satisfaction coefficient ratio $\;RI_{i}$	Classification
X_1_	29.3	54.9	11.0	4.9	0.0	0.40	-0.66	0.61	M
X_2_	9.8	31.7	17.1	40.2	1.2	0.27	-0.49	0.55	I
X_3_	13.4	47.6	26.8	12.2	0.0	0.40	-0.74	0.54	M
X_4_	9.8	52.4	13.4	24.4	0.0	0.23	-0.66	0.35	M
X_5_	31.7	41.5	19.5	7.3	0.0	0.51	-0.61	0.84	M
X_6_	29.3	24.4	34.1	9.8	2.4	0.65	-0.60	1.08	O
X_7_	39.0	12.2	31.7	17.1	0.0	0.71	-0.44	1.61	A
X_8_	25.6	3.7	19.5	47.6	3.7	0.47	-0.24	1.95	I
X_9_	26.8	20.7	45.1	6.1	1.2	0.73	-0.67	1.09	O
X_10_	42.7	12.2	18.3	24.4	2.4	0.63	-0.31	2.00	A
X_11_	26.8	18.3	42.7	12.2	0.0	0.70	-0.61	1.14	O
X_12_	14.6	3.7	17.1	62.2	2.4	0.33	-0.21	1.53	I
X_13_	59.8	3.7	18.3	18.3	0.0	0.78	-0.22	3.56	A
X_14_	48.8	0.0	4.9	46.3	0.0	0.54	-0.05	11.00	A
X_15_	19.5	13.4	20.7	45.1	1.2	0.41	-0.35	1.18	I
X_16_	22.0	11.0	46.3	20.7	0.0	0.68	-0.57	1.19	O
X_17_	29.3	3.7	32.9	34.1	0.0	0.62	-0.37	1.70	I
X_18_	33.8	15.0	17.5	32.5	1.3	0.52	-0.33	1.58	A
X_19_	40.5	23.8	3.6	28.6	3.6	0.46	-0.28	1.61	A
X_20_	45.1	18.3	25.6	8.5	2.4	0.73	-0.45	1.61	A
X_21_	29.3	13.4	12.2	36.6	8.5	0.45	-0.28	1.62	I
X_22_	25.6	52.4	13.4	8.5	0.0	0.39	-0.66	0.59	M
X_23_	18.3	6.1	32.9	41.5	1.2	0.52	-0.40	1.31	I
X_24_	35.4	22.0	29.3	13.4	0.0	0.65	-0.51	1.26	A
X_25_	30.5	4.9	17.1	43.9	3.7	0.49	-0.23	2.17	I
X_26_	0.0	3.7	0.0	26.8	69.5	0.00	-0.12	0.00	R
X_27_	23.2	22.0	18.3	35.4	1.2	0.42	-0.41	1.03	I
X_28_	8.5	28.0	15.9	45.1	2.4	0.25	-0.45	0.56	I
X_29_	3.7	12.2	18.3	50.0	15.9	0.26	-0.36	0.72	I

Due to the lengthy experimental process and high requirements for the cooperation of the children, after multiple communications, 105 children with ASD were ultimately selected as the experimental group. And provide detailed information on the content, purpose, and significance of this experiment to the parents of the children being tested, seek their consent, and obtain a voluntary informed consent form signed by the parents. The provided informed consent form is complete in content, and the risk disclosure is objective, sufficient, and the method and process of obtaining individual informed consent are compliant and appropriate. Conducting research using anonymized information data by observing and not interfering with data generated from public behavior. Not causing harm to the human body, not involving sensitive personal information or commercial interests. The source of the biological samples used complies with relevant regulations and ethical principles, and the research content and purpose are within the scope of standardized informed consent.

**Figure 6:**
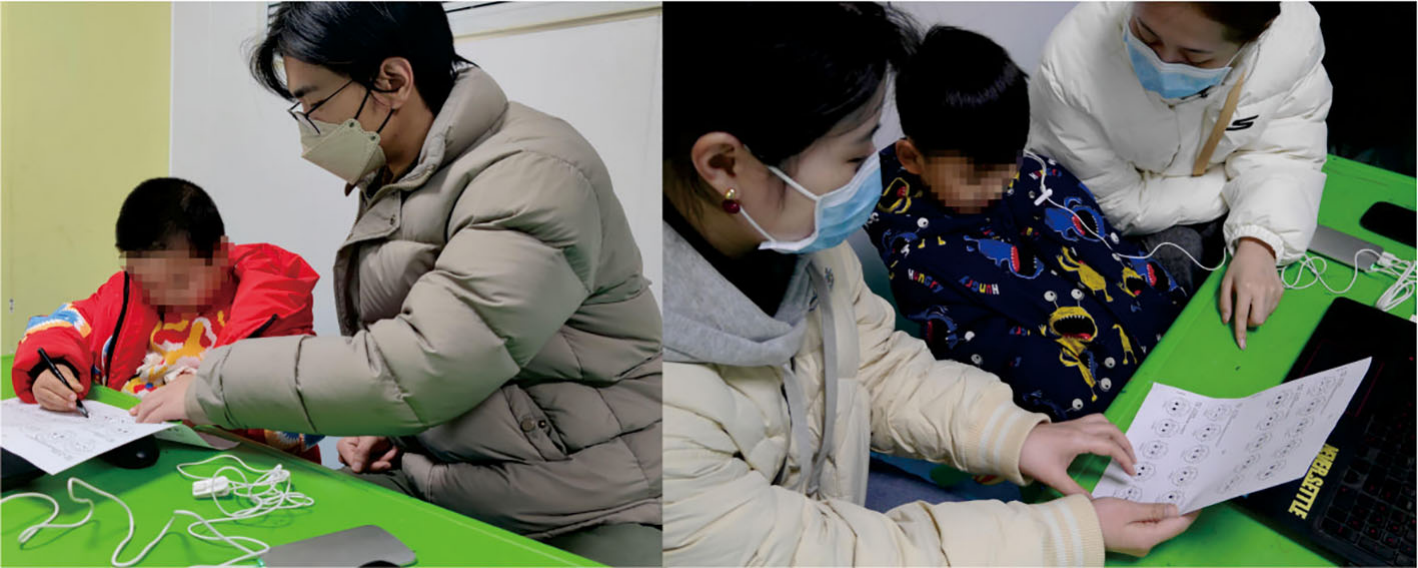
Experiment scene.

The specific situation of the children involved is shown in **Table 7**. This process invites parents or teaching assistants of the participants to provide assistance in order to handle unexpected situations at any time; During the experiment, invite the parents or teaching assistants of the children to provide assistance in order to handle any unexpected situations at any time; After the experiment, the children were given rewards such as candy and picture books. The experimental scene is shown in **Figure 6**. Due to experimental limitations, further detailed analysis of gender differences was not conducted.

**Table 7:** Basic introduction of the subjects.

	Number of people	Girls	Boys
4∼5 years	41	12	29
6∼7 years	64	23	41

It can be seen from **Table 4** that user requirements X2,X8,X12,X15,X17,X21,X23,X25,X27,X28 and X29 are indifferent requirements, which will not affect the design of the intervening APP interface and should be eliminated; user requirements X26 This is a reverse demand, which may be due to the protection of the privacy of ASD children, teaching assistants and parents are unwilling to disclose children’s information, and the provision of personal platform push functions will lead to a decrease in satisfaction, so it is removed.

### 4.4 Entropy method to calculate the weight of demand indicators

The expert group was invited again to evaluate the importance of 8 exciting needs, 5 basic needs and 4 expected needs. In the process, a five-level Likert scale was used and a decision matrix was constructed. The evaluation results are shown in **Table 8**.

**Table 8:** Evaluation results of user demand importance.

Numbering	X_1_	X_3_	X_4_	X_5_	X_6_	X_7_	X_9_	X_10_	X_11_	X_13_	X_14_	X_16_	X_18_	X_19_	X_20_	X_22_	X_24_
Teaching Assistant1	5	5	4	4	5	3	5	5	5	5	3	4	3	3	5	5	3
Teaching Assistant2	4	4	5	5	4	3	3	2	4	3	2	5	2	3	3	4	4
Teaching Assistant3	5	4	4	3	5	5	4	4	5	4	5	4	4	5	3	4	1
Teaching Assistant4	4	3	4	4	4	3	3	5	4	3	4	4	3	4	4	4	2
Teaching Assistant5	3	4	5	3	4	4	5	3	5	5	2	4	4	4	3	5	2
Teaching Assistant6	5	4	4	5	5	3	4	5	4	5	3	4	2	4	4	5	2
Teaching Assistant7	5	5	4	3	4	5	3	4	5	3	3	5	2	3	3	4	3
Teaching Assistant8	4	4	5	4	5	4	4	2	5	3	4	5	3	5	3	4	3
Teaching Assistant9	5	4	4	5	4	4	4	3	4	3	3	5	3	5	3	4	1
Designer1	5	3	3	3	5	3	4	4	5	5	2	4	4	3	4	4	2
Designer2	4	5	4	5	5	4	4	3	4	4	4	5	3	4	4	4	4
Designer3	4	4	4	5	4	4	3	2	5	5	3	4	3	4	4	5	3

Then the decision matrix is standardized according to Equation 3, and a standardized matrix for evaluating the importance of user needs of the intervention APP interface is obtained. Then Equations 4, 5 can calculate the entropy value of each user demand index; on this basis, Equation 6 can calculate the weight value of each user demand index, as shown in **Table 9**. Show.

**Table 9:** The weight of user demand index of intervention app interface.

Kano demand type	Numbering	Weights	Sort	Kano demand type	Numbering	Weights	Sort
basic requirements	X_1_	0.03420	17	Exciting requirements	X_7_	0.06598	5
	X_3_	0.04111	15		X_10_	0.05054	12
	X_4_	0.03433	16		X_13_	0.06471	6
	X_5_	0.05601	10		X_14_	0.05079	11
	X_22_	0.10113	1		X_18_	0.04857	13
Expected requirements	X_6_	0.07279	4		X_19_	0.05685	8
	X_9_	0.05646	9		X_20_	0.07586	3
	X_11_	0.06201	7		X_24_	0.04313	14
	X_16_	0.08553	2				

## 5 Results

### 5.1 Analysis of the weight of demand indicators

By sorting out the weights of the demand indicators obtained from the experiment, combined with relevant literature research (47–49), the indicators can be divided into three types according to their main characteristics.

1. The content requirements are mainly related to the functional content and intervention of the APP. This includes high recognition of the content interface, use in family settings, high attractiveness of the content interface, rich levels of content, diverse reward mechanisms, and operational history.

2. The operational requirements are mainly related to the operating mechanism of the intervention APP. Including good operational feedback, moderate jumping speed, voice interaction function, simple and convenient operation, and customization of personalized features.

3. Visual needs are mainly related to the visual information encoding form of the intervention APP interface. Including a well-structured layout, concise and intuitive information, vivid images, clear navigation, rich textual elements, and interesting fonts.

It can be seen that content needs are studied from the perspective of the content mode of intervention apps and the audience of ASD children. Based on the visual cognitive characteristics of ASD children, interface content that can attract children’s sustained attention is designed. Operational requirements refer to the study of the operational mechanism of intervening in the APP interface, based on the user experience of the intervention APP, to make the design conform to the user’s operational behavior pattern. Visual requirements refer to the study of various visual elements such as text, color, images, and videos, and the design of effective interface layout, navigation hierarchy, color matching, and icons based on the graphic indication and guidance of the APP interface.

According to the classification and sorting, the weight order of the three categories of needs for intervention APP is: content needs>visual needs>operational needs, that is, children with ASD have the highest demand for content indicators (see **Table 10**). Therefore, in the design process, content requirements can be considered as the most critical core content.

**Table 10:** Demand index classification.

Demand category	Numbering	Weights
Content requirements	X_6_,X_14_,X_16_,X_19_,X_20_,X_22_	0.44294
Operational requirements	X_3_,X_4_,X_7_,X_9_,X_18_	0.24645
Visual requirements	X_1_,X_5_,X_10_,X_11_,X_13_,X_24_	0.31061

1. Among the content demand categories, the three indicators of “operation history”, “high content attractiveness” and “rich reward mechanism” have the highest demand weight values. In the design process, a more convenient way to save, manage and check the operation history, improve and enrich the reward mechanism, and continuously enhance the attractiveness of the interface content, in order to increase the learning interest of ASD children.

2. In the visual demand category, the demand weights of “clear and clear navigation” and “rich character elements” are higher. The visual effect design of the APP interface can directly affect the user’s first impression of the product and the user’s emotions. Compared with other ways of obtaining information, visual information is more likely to attract the attention of ASD children (50). Therefore, in the design process, it is necessary to make a reasonable plan for the visual information of the interface, enrich the character elements, and provide a clear and guiding navigation design to avoid interference information.

3. In the category of operational requirements, “voice interactive function” and “easy and convenient operation” have higher demand weight values. The APP interface design should meet the user’s needs for product ease of use and conform to the user’s cognitive habits for quick operation (51). In the design process of intervening APP interface operation functions, simple and effective operation methods and voice interaction functions should be provided for ASD children to reduce learning time and enhance the interactive experience.

Scholar LI S divides the evaluation image of communication APP interface design into three dimensions (13), among which aesthetics is more similar to the visual requirements in this article, usability and complexity are more related to the content requirements and operational requirements in this article, but focus more on subjective descriptions of individuals. The dimensions of this study are more easily reflected in the refinement of indicators in design practice.ZHOU Y regards shape cognition and reward mechanism as the main basis for the design practice part (14), and this article attributes these two indicators to the more influential expected requirements and exciting requirements, reflecting a relatively consistent viewpoint.Several scholars believe that in the communication and intervention training process for children with ASD, the intervention of visual cognition and familiar groups, including teaching assistants, parents, etc., can effectively improve the effectiveness, which is very similar to the methods and approaches adopted in this study (15, 17, 20). Jian L extracted 7 aesthetic indicators as measurement indicators to interpret quality characteristics and established a layout aesthetic evaluation model for information interface quality (18). Among them, orderliness, complexity, guidance, and consistency are very close to the main indicators covered by the user needs proposed in the article.WANG believes that improvements in form and color appeal principles can meet visual needs, while effective functionality and human-machine comfort principles can meet content and operational requirements, which is closer to this study (30). YU Guang Chen believes that styling and beauty belong to exciting requirements, while feedback on sound inspection, anti-collision, and other operations belong to basic requirements (31), which is similar to this study. However, expected requirements differ in that they do not include a good user experience, but instead add auxiliary functions such as sleep and educational assistance. Yi Xuan W believes that operational requirements such as easy operation have a good effect on improving user experience; The visual requirements also have a significant impact on browsing efficiency, navigation functionality, and other visual effects (36), but multiple indicators included in the content requirements listed in this article, such as reward mechanisms and operation records, were not taken into account.

Fu Han has clearly proposed four basic criteria for interface design: visual requirements, interactive requirements, functional requirements, and emotional requirements. The first three have a lot of consistency with the classification of visual requirements, content requirements, and operational requirements in this article (38). However, emotional requirements were not included as a separate indicator in this article because it is considered that emotional recognition in children with ASD has certain weaknesses. It is hoped that special discussions can be conducted in the future. Jinsong Huang believes that extracting user appearance requirements, functional requirements, operational requirements, development requirements, and security requirements, totaling 5 items, is an important part of new product development (39). The first four categories are consistent with this study, but considering that development requirements are scattered among various requirements and security requirements are relatively few, this study has not yet proposed them. Deng Zhao believes that in the design process, functional indicators should be included in basic requirements, color and visual cues should be included in expected requirements, and new experiences should be included in exciting requirements, which is consistent with the framework of this study (41). LI Saisai classifies and analyzes the demand indicators of logistics systems from a multi-user perspective, rather than a single user, such as doctors, patients, nurses, manufacturers, technical maintenance personnel, etc. (42), which is different from most known scholars, but also provides a reference for further in-depth research.

It can be seen that the demand classification and indicator extraction conducted in this study are consistent with many existing research viewpoints, and some aspects that are not completely consistent or clear enough need further in-depth research and verification in the future.

### 5.2 Design case

**Figure 7:**
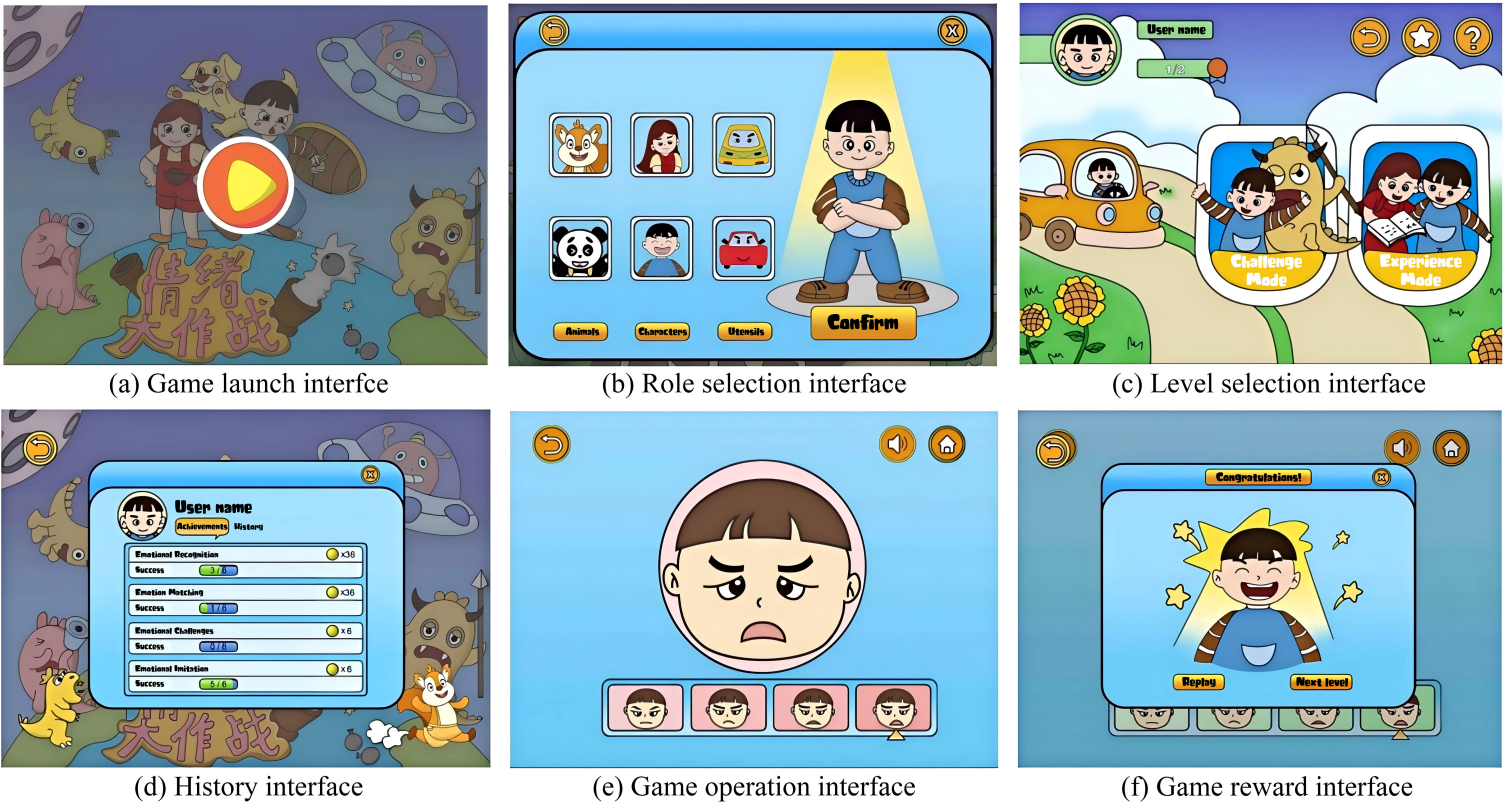
Design case of ASD children intervention APP interface [including **(a)** game launch, **(b)** role selection, **(c)** level selection, **(d)** history, **(e)** game operation, and **(f)** reward interfaces].

On the basis of dividing the demand indicators into three types: content demand, operational demand and visual demand, combined with ASD children’s color aesthetic preference (52, 53), the theme of improving emotional ability is used for related interface design, such as Shown in **Figure 7**.

1. First of all, in response to content requirements, when the game is over, the operation history record function is designed, which can record in detail the children’s training level of emotion recognition, matching, challenge, and imitation, so that parents can understand the children’s growth dynamics and intervention training results (**Figure 7d**). Taking into account the visual cognitive characteristics of ASD children, the interface background uses elements such as monsters, spaceships, children, and the earth to enrich the interface background and increase its attractiveness (**Figure 7a**). In addition, after each level is over, a variety of reward mechanisms are designed to stimulate children’s interest in learning and enhance the effect of intervention training (**Figure 7f**).

2. For the visual requirements, a large number of animals, characters and utensils are used in the interface design, which is lively and vivid, and draws children’s closeness to the interface. A white border is used to assist in the selection of roles and levels, which can highlight important content, arouse children’s curiosity, prompt them to click on the screen, and enhance the frequency of human-computer interaction (**Figures 7b, c**).

3. Finally, there are operational requirements. There is a voice broadcast function before the start of the game, during use, and after the end of the game, to guide children to the next step. The navigation buttons are located at both ends of the interface. Simple graphic symbols are used, such as arrows to indicate return, cross means closed, house means home page, etc., with yellow as the bottom, clear and obvious, and strong in guidance (**Figure 7e**).

When developers apply models, they need to pay attention to the effectiveness of communication during the process of collecting and researching user needs, such as using the help of teaching assistants, parents, and other personnel to obtain and filter effective demand information.Before conducting indicator weight analysis, it is necessary to ensure that the evaluation is as clear and accurate as possible. Experienced industry experts can be hired to provide a solid foundation for the application of the model.When using models for visual presentation, it is necessary to adopt more effective styling designs as much as possible, such as referring to some visual forms that children prefer, such as popular picture books, cartoons, and toys.

## 6 Discussion

In summary, enhancing the content elements of Attractive demand can motivate children with ASD to gain sustained attention and effectively increase their attention to the content during the actual interaction process, which can be realized by continuously enriching the content of the APP to improve the attractiveness, perfecting the reward mechanism, increasing the dynamic information, improving the continuity of the intervention training, and lengthening the training time, etc. In this regard, we have developed a new approach for the development of visual demand.

Expanding the visual One-dimensional demand can gather the attention of children with ASD and provide smooth intervention training. This can be achieved by adding interesting fonts and cartoon characters in the APP, enriching the text elements, enhancing the fun and stimulating the children’s interest, and reducing the children’s anxiety and irritability through a good visual experience as much as possible.

Extending the Must-be operation requirements can enhance the real-time interactive feedback and bring a more efficient and pleasant interactive experience. Attention should be paid to avoiding vague instructions in the content of the APP to increase children’s operational convenience, and reasonable function layout and voice interaction can enhance operational convenience, improve user satisfaction, and establish children’s loyalty to the APP.

However, it is worth noting that inappropriate stacking of demand elements may lead to visual interference or information overload, aggravating the tension and discomfort of children with ASD, and the designer still needs to optimize the ratio of the three demands and the level and style of the visual presentation through multiple rounds of iteration and user feedback, so that it is attractive enough without being overly stimulating.

This paper integrates the Kano model and the entropy method to design and research the ASD child intervention APP interface, and conducts statistical analysis of user needs based on expert interviews, tries to combine subjective experience with objective quantification, and further improves the interface design method of intervention APP.

**Figure 8:**
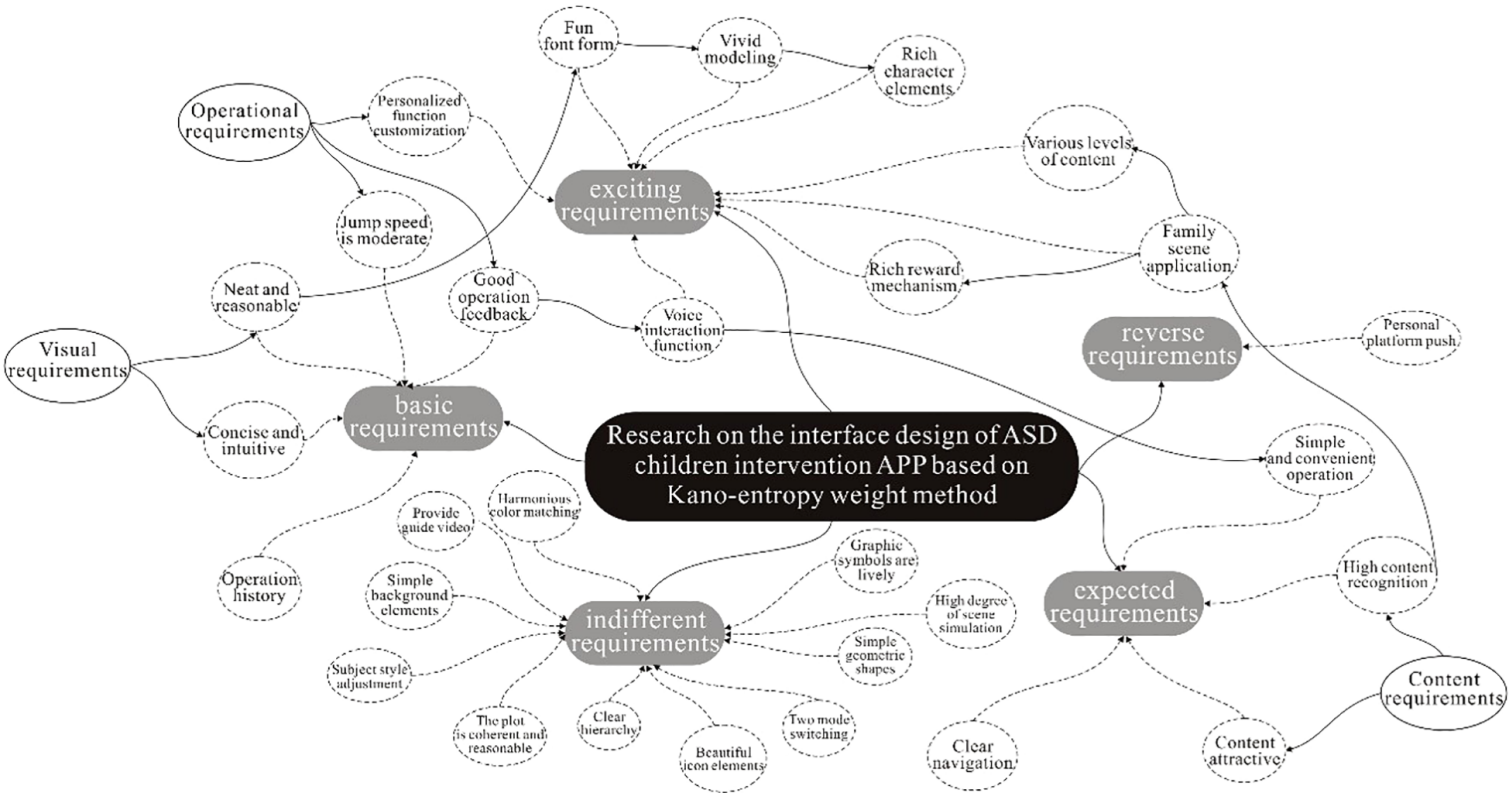
Interface design method of ASD children intervention APP.

On the basis of organizing 26 pieces of user demand information, the Carnot model was used to extract 17 specific indicators related to APP design, including basic needs, expected needs, and incentive needs, as shown in **Table 9**. On this basis, a refined demand index entropy method is used to calculate weights and rank them; Based on the indicators and training relevance of ASD children, the 17 indicators are divided into three categories: content based, operability based, and visual based, as shown in **Table 10**. The key points of the interface design method for the intervention application for children with autism are ultimately formed, as shown in **Figure 8**.

It can be seen that the Kano-entropy method, which combines subjective evaluation and objective quantification, can more clearly and intuitively analyze the characteristics of ASD children’s needs, further enrich the content of ASD children’s intervention APP, improve operations, and enhance visual appeal, thereby obtaining training The improvement of effect provides reference and basis for subsequent related design and enhances the effectiveness of intervention training. Based on the above research, in the future, visual cue strategies can be linked to the icons and colors of ASD children’s intervention app interfaces, and combined with gender differences in children, for more systematic and in-depth research.

The research experiments conducted by our institute were mainly conducted in Xuzhou, Jiangsu Province, China, and have certain regional limitations. I hope to increase the number of participants across regions in future research to broaden user coverage.The design of the intervention app involves cross disciplinary, multi-disciplinary, and fusion technologies. We hope to conduct more comprehensive design research in future studies and develop a software that can be put into use.Differences in user age, gender, family background, and other factors can have an impact on demand. If conditions permit, it is hoped that more comprehensive research can be conducted on different segmented user groups in the future to make experimental data more accurate and valuable for reference.

## Data Availability

The original contributions presented in the study are included in the article/supplementary material. Further inquiries can be directed to the corresponding author.
